# Protective Effects of Sporoderm-Broken Spores of *Ganderma lucidum* on Growth Performance, Antioxidant Capacity and Immune Function of Broiler Chickens Exposed to Low Level of Aflatoxin B_1_

**DOI:** 10.3390/toxins8100278

**Published:** 2016-09-24

**Authors:** Tao Liu, Qiugang Ma, Lihong Zhao, Ru Jia, Jianyun Zhang, Cheng Ji, Xinyue Wang

**Affiliations:** 1 State Key Laboratory of Animal Nutrition, College of Animal Science and Technology, China Agricultural University, Beijing 100193, China; liuyitao2004@126.com (T.L.); lihongzhao100@126.com (L.Z.); jyzhang@cau.edu.cn (J.Z.); jicheng@cau.edu.cn (C.J.); xinyuewang2016@126.com (X.W.); 2 College of Life Science, Shanxi University, Taiyuan 030006, Shanxi, China; jiaru.bjdk071@163.com

**Keywords:** spores of *Ganoderma lucidum*, oxidative stress, aflatoxins, antioxidant capability

## Abstract

This study was conducted to investigate the toxic effects of aflatoxin B_1_ (AFB_1_) and evaluate the effects of sporoderm-broken spores of *Ganoderma lucidum* (SSGL) in relieving aflatoxicosis in broilers. A total of 300 one-day-old male Arbor Acre broiler chickens were randomly divided into four dietary treatments; the treatment diets were: Control (a basal diet containing normal peanut meal); AFB_1_ (the basal diet containing AFB_1_-contaminated peanut meal); SSGL (basal diet with 200 mg/kg of SSGL); AFB_1_+SSGL (supplementation of 200 mg/kg of SSGL in AFB_1_ diet). The contents of AFB_1_ in AFB_1_ and AFB_1_+SSGL diets were 25.0 μg/kg in the starter period and 22.5 μg/kg in the finisher period. The results showed that diet contaminated with a low level of AFB_1_ significantly decreased (*p* < 0.05) the average daily feed intake and average daily gain during the entire experiment and reduced (*p* < 0.05) serum contents of total protein IgA and IgG. Furthermore, a dietary low level of AFB_1_ not only increased (*p* < 0.05) levels of hydrogen peroxide and lipid peroxidation, but also decreased (*p* < 0.05) total antioxidant capability, catalase, glutathione peroxidase, and hydroxyl radical scavenger activity in the liver and spleen of broilers. Moreover, the addition of SSGL to AFB_1_-contaminated diet counteracted these negative effects, indicating that SSGL has a protective effect against aflatoxicosis.

## 1. Introduction

Aflatoxins (AFBs) are the most common mycotoxins and are mainly produced by *Aspergillus flavus* and *Aspergillus parasiticus* [1]. Among all the AFBs, aflatoxin B_1_ (AFB_1_) is the most highly toxic contaminant in foods and feedstuffs, and is classified by the International Agency of Research on Cancer (IARC) as a Group 1 carcinogen. In poultry, consumption of AFB_1_ can cause huge economic losses by retarding animal growth, increasing feed efficiency and the incidence of disease, and inducing damage to organs such as the liver and spleen [2,3]. Moreover, the carry-over of AFB_1_ through animal-derived products into the human food chain is a potential threat to human health due to its hepatotoxicity, carcinogenicity, mutagenicity, teratogenicity, and immunosuppression [4,5]. AFB_1_ is the most widespread oxidative agent of the AFBs [6], and it was reported that the toxic effects of AFB_1_ were intimately linked with its pro-oxidant which could induce the generation of reactive oxygen species (ROS) and lead to the impairment of DNA, RNA, proteins, lipids, and other molecules [7,8]. Therefore, some studies suggested that the addition of antioxidants to diets could protect animals against AFB_1_-induced toxicity by enhancing the antioxidant system and immunity [9,10].

*Ganoderma lucidum* (*G. lucidum*), a traditional folk medicinal mushroom, has been used as an important longevity and health-promoting herb for more than 2000 years in China [11]. The spores of *Ganoderma lucidum* (SGL), ejected from the pileus of *G. lucidum* in the mature phase, are tiny and mist-like particles of about 6.5–8.0 × 9.6–12.6 μm enwrapped with an outer bilayer of sporoderm [12]. During the past two decades, because the spores could be collected on a large scale, SGL has attracted extensive interest as studies revealed that the spores possess many bioactive substances, including polysaccharides, unsaturated fatty acids, triterpenoids, nucleotides, ergostero and other bioactive ingredients [13,14]. In vitro, the polysaccharides of SGL had strong bioactivity in scavenging 1,1-Diphenyl-2-picrylhydrazyl and oxygen radicals [15]. In addition, antioxidant activities of polysaccharides from SGL were also demonstrated in rats [16]. Furthermore, the polysaccharides from SGL had potent stimulating effects on spleen mass, lymphocyte proliferation and antibody production in mice [17,18]. Fatty acids are another kind of potential active ingredient of SGL, which showed high bioactivities in antitumor and immunomodulation in rats [19,20]. However, the bioactivities of spores are closely related to the status of the sporoderm; the intact sporoderm can inhibit the release and absorption of bioactive substances in the spores [21]. Yue et al. [22] observed that dietary sporoderm-broken spores of *Ganoderma lucidum* (SSGL) were more effective in stimulating the production of interferon-γ, interleukin-2, interleukin-4 and interleukin-6 in mice than dietary sporoderm-unbroken spores of *G. lucidum*. Therefore, we speculated that supplementation of SSGL to AFB_1_-contaminated diets might alleviate aflatoxicosis through decreasing the oxidative status and elevating the antioxidant defense system.

Our previous study showed that different levels of SSGL (100, 200 and 500 mg/kg) improved the average daily gain, decreased the feed:gain ratio, and enhanced the antioxidant capacity in the liver and spleen of broilers. In this study, SSGL was chosen to evaluate its protective effects on growth performance, antioxidant function and serum immunoglobulins of male Arbor Acre broilers exposed to a low level of AFB_1_.

## 2. Results

### 2.1. Effects of SSGL on Growth Performance of Male Arbor Acre Broiler Chickens Exposed to Low Level of AFB_1_

In 0–21 d, broilers fed a diet contaminated with AFB_1_ resulted in a significant decrease (*p* < 0.05) in average daily feed intake (ADFI) as compared to the control group (Table 1). The addition of SSGL into the diet contaminated with AFB_1_ significantly increased (*p* < 0.05) the ADFI of broilers as compared to the AFB_1_ group. The ADFI of broilers among control, SSGL and AFB_1_+SSGL groups showed no significant differences (*p* > 0.05). The average daily gain (ADG) and feed:gain ratio (F:G) were not significantly affected (*p* > 0.05) by a low level of AFB_1_ or dietary SSGL in broilers.

In 22–44 d, broilers in the AFB_1_ group had significantly lower (*p* < 0.05) ADFI and ADG than those in the control group. However, supplementation of SSGL to the AFB_1_-contaminated diet obviously increased (*p* < 0.05) ADFI, ADG and decreased (*p* < 0.05) F:G as compared with the AFB_1_ group. In addition, broilers in the SSGL and AFB_1_+SSGL groups had significantly higher (*p* < 0.05) ADG than those in the control group, but ADFI, ADG and F:G between the SSGL and AFB_1_+SSGL groups showed no significant differences (*p* > 0.05). Similar effects of SSGL on the growth performance (ADG, ADFI and F:G) of broilers exposed to a low level of AFB_1_ were also observed during the entire experiment (0–44 d).

### 2.2. Effects of SSGL on Oxidative Status in Liver and Spleen of Male Arbor Acre Broiler Chickens Exposed to Low Level of AFB_1_

The data in Table 2 showed that the hepatic levels of H_2_O_2_, MDA and LPO of broilers in the AFB_1_ group were significantly higher (*p* < 0.05) than those in the control group, but these indexes were markedly decreased (*p* < 0.05) by the supplementation of SSGL into the diet contaminated with AFB_1_. Moreover, broilers in the SSGL group had significantly lower (*p* < 0.05) hepatic levels of H_2_O_2_ and MDA than those in the control group. A higher (*p* < 0.05) hepatic H_2_O_2_ level and lower (*p* < 0.05) hepatic MDA level of broilers were observed in the AFB_1_ group as compared to the control group, but the hepatic LPO level of broilers showed no difference (*p* > 0.05) among the control, SSGL and AFB_1_+SSGL groups.

Similar to the liver, the splenic levels of H_2_O_2_ and LPO of broilers in the AFB_1_ group were significantly increased (*p* < 0.05) as compared with those in the control group (Table 2). However, the addition of SSGL in the diet contaminated with AFB_1_ significantly reduced (*p* < 0.05) H_2_O_2_ and LPO levels in the spleen compared with the AFB_1_ group. In addition, the splenic level of LPO in the SSGL and AFB_1_+SSGL groups was significantly lower (*p* < 0.05) than that in the control group, and broilers in the SSGL group had a lower (*p* < 0.05) MDA level than those in AFB_1_ group. These results showed that diets contaminated with AFB_1_ could aggravate the oxidative status of broilers, which could be relieved by the supplementation of SSGL.

### 2.3. Effects of SSGL on Antioxidant Defense System in Liver and Spleen of Male Arbor Acre Broiler Chickens Exposed to Low Level of AFB

Broilers fed a diet contaminated with AFB_1_ had significantly decreased (*p* < 0.05) T-AOC, CAT, GR, GSH-Px and HRSA levels in the liver as compared with those in the control group (Table 3), while the addition of SSGL into the diet contaminated AFB_1_ significantly enhanced (*p* < 0.05) the levels of T-AOC, CAT, GR, HRSA and GSH in the liver. When compared to the control group, hepatic enzyme activities (T-AOC, CAT, GR) and the GSH level of broilers were significantly increased (*p* < 0.05) in the SSGL group, whereas a significantly lower (*p* < 0.05) hepatic activity of GSH-Px and a higher (*p* < 0.05) level of GSH were observed in broilers in the AFB_1_+SSGL group. The hepatic activity of T-SOD of broilers exposed to AFB_1_ was not significantly affected (*p* > 0.05) by dietary supplementation with SSGL.

Similar negative effects of AFB_1_ and ameliorative effects of dietary SSGL on the antioxidant defense system were also found in the spleen (Table 3). The splenic levels of T-AOC, CAT, T-SOD, GSH-Px, HRSA and GSH of broilers in the AFB_1_ group were lower (*p* < 0.05) than those in the control group. However, supplementation of SSGL to the AFB_1_-contaminated diet significantly enhanced (*p* < 0.05) splenic levels of T-AOC, T-SOD, GR, HRSA and GSH as compared to the AFB_1_ group. In addition, broilers in the SSGL group had significantly higher T-AOC, GR, GSH, HRSA levels than those in the control group, and significantly lower (*p* < 0.05) activities of CAT, GSH-Px, and higher (*p* < 0.05) activities of GR, HRSA and GSH were observed in the AFB_1_+SSGL group as compared to the control group. The splenic activities of T-AOC, CAT, GR, GSH-Px, HRSA in the SSGL group were higher (*p* < 0.05) than those in the AFB_1_+SSGL group.

### 2.4. Effects of SSGL on Serum Total Protein and Immunoglobulins of Male Arbor Acre Broiler Chickens Exposed to Low Level of AFB_1_

The contents of serum TP, IgA and IgG of broilers exposed to a low level of AFB_1_ were significantly lower (*p* < 0.05) than those in the control group (Table 4), while these indexes were significantly improved by the addition of SSGL to the diet contaminated with AFB_1_. However, the contents of serum TP, IgA and IgG of broilers among the control, SSCL and AFB_1_+SSGL groups showed no significant differences (*p* > 0.05). There was no significant difference (*p* > 0.05) in the serum IgM of broilers among all the groups.

## 3. Discussion

### 3.1. Toxicity of AFB_1_ on Growth Performance

Feedstuffs are easily contaminated with AFB_1_ during harvesting, transportation and storage, and it was reported that the detection rates of AFB_1_ were 50% in corn, 36% in soybean meal, 94% in DDGS, respectively, in the Beijing region [23], and 15.39% in peanuts in the south of China [24]. Therefore, the presence of AFB_1_ in animals’ diets is hard to avoid, and it is increasingly recognized that the long-term consumption of low levels of AFBs is detrimental to animals’ growth and health. In this study, diet contaminated with AFB_1_ caused a significant decrease in the ADFI of broilers during the starter period (0–21 d), and not only the ADFI but also the ADG of broilers during the finisher period (22–44 d) were significantly reduced by dietary AFB_1_. Similar results had been observed in the previous studies, in which an AFB_1_-containing diet (50–100 μg/kg) could significantly decrease the body weight gain and feed consumption of broilers, causing economic loss [2,25]. Ducks fed diets containing 20 μg/kg of AFB_1_ had a significantly lower ADG, ADF, and higher F:G [26]. These results indicated that diets containing a low level of AFB_1_ (22.5–25.0 μg/kg) could inhibit broiler growth and cause great financial losses.

### 3.2. Toxicity of AFB_1_ on Oxidative Stress of Broilers

Liver and immune system organs such as the spleen are considered to be sensitive to AFB_1_ [27,28]. It had been reported that the toxic effect elicited by AFB_1_ could be closely related to the generation of reactive oxygen species (ROS), mainly including superoxide (O_2_^−^), hydrogen peroxide (H_2_O_2_), and hydroxyl (OH^·^) [29]. H_2_O_2_ is released from the mitochondria; hydroxyl radicals (OH^·^) are also an extremely reactive free radical. Biosynthesis and accumulation of ROS are central to oxidative stress-related metabolism [30]. The exceeded ROS could enhance lipid peroxidation, which will impair membrane function by decreasing membrane fluidity and changing the activities of membrane-bound enzymes and receptors [31]. MDA is formed at the end of lipid peroxidation, and reflects the degree of the whole lipid oxidation in the body. In this study, the contents of H_2_O_2_, MDA and LPO in the liver and spleen of broilers were significantly increased by dietary AFB_1_, indicating that a low level of AFB_1_ (22.5–25.0 μg/kg) could increase the oxidative status in the liver and spleen of broilers. Similar toxic effects of AFB_1_ on oxidative status were observed in the liver and spleen of rats [32], and broilers [33,34].

The levels of free radical molecules and lipid peroxidation are controlled by an antioxidant defense system, consisting of enzymatic components such as SOD, CAT, and GR, and non-enzymatic components such as GSH and vitamin E [35]. When oxidative stress arises as a pathologic event, the defense system will promote the regulation and expression of enzymatic and non-enzymatic components [36]. SOD can detoxicate O_2_^−^ to H_2_O_2_, and then GSH-Px and CAT catalyze H_2_O_2_ directly to water and oxygen at the expense of GSH [37,38]. Many studies observed that dietary AFB_1_ could decrease the activities of antioxidant enzymes and levels of non-enzymatic antioxidants in broilers [9,39,40]. In the present study, the antioxidant enzyme activities and GSH level in the liver and spleen of broilers fed a diet contaminated with AFB_1_ were significantly lower than those in the control group, suggesting that the diet contaminated with a low level of AFB_1_ (22.5–25.0 μg/kg) could suppress the antioxidant capacity of broilers.

### 3.3. Toxicity of AFB_1_ on Serum Total Protein and Immunoglobulins of Broilers

Except for the toxic effects of AFB_1_ on the liver and spleen, the immunosuppression in animals is also a matter of concern, because it may predispose farm animals to infectious diseases and result in economic losses [41]. Immunoglobulins, secreted by B cells, are the main antibody isotypes of the serum and extracellular fluid immune system, thereby allowing them to control the infection of body tissues. AFB_1_ is known to be immunosuppressive in birds, and it has been reported that diets containing 300 μg/kg of AFB_1_ significantly reduced the serum IgA, IgG and IgM of broilers [42]. In addition, chickens fed aflatoxin at a concentration of 2500 μg/kg had lower serum total protein (TP), albumin and IgG, but serum IgM was not affected by AFBs [43]. Serum TP is the indicator of protein synthesis, and the decreased serum TP induced by dietary AFB_1_ may contribute to the decreased contents of immunoglobulins [44,45]. In the present study, broilers fed diets contaminated with a low level of AFB_1_ (22.5–25.0 μg/kg) had significantly lower serum TP, IgA and IgG, while serum IgM was not significantly affected by dietary AFB_1_. One mechanism of action of AFBs is related to the inhibition of protein synthesis, which may be responsible for the decrease in serum IgA and IgG in this study.

### 3.4. Effects of SSGL

Seeking effective ways to alleviate the negative effects of AFB_1_ has attracted more and more attention. Nowadays, researchers found that different feed additives had the ability to relieve aflatoxicosis. Alpha-lipoic acid, known as an “ideal antioxidant”, could prevent hepatic oxidative stress and down-regulate the expression of hepatic pro-inflammatory cytokines of broilers exposed to a low level of AFB_1_ [46]. Selenium, playing important roles in immune function, could protect chickens from AFB_1_-induced impairment of humoral and cellular immune function by reducing bursal histopathological lesions and percentages of apoptotic bursal cells [47]. Vitamin E, as an antioxidant, could ameliorate AFB_1_-induced toxicity in rats [9] and in ducks [48]. Moreover, some plants or their extracts, such as Chinese cabbage powder [49] and tinospora cordifolia root extract [50], were demonstrated to counteract the detrimental toxic effects of AFBs. Although SGL, as a traditional Chinese herb, is characterized as having antioxidation, immunomodulation and hepatoprotection activities, there is no study to assess the protective effects of SGL or its extracts on AFB_1_-induced toxins in broiler chickens. In the present study, the results showed that the addition of SSGL to an AFB_1_-contaminated diet relieved the negative effects of AFB_1_ on the growth performance of broilers.

SGL has strong bioactivities in antioxidation, and polysaccharides from SGL had been reported to have the ability to scavenge DPPH radicals, increase reducing power and inhibit lipid peroxidation in vitro [51]. Moreover, SGL was effective in protection against Cd(II)-induced hepatotoxicity through reducing the increase in serum AST, ALT and hepatic MDA of mice [52]. Supplementation with 4 or 8 g/kg of SGL significantly increased the activities of GSH and GR, and decreased the MDA content in the hippocampus of rats [15]. In type 2 diabetic rats, the serum level of MDA was 13.9% lower, and serum levels of GSH-Px and SOD were 25.9% and 38.0% higher at four weeks in the SGL group [53]. Some antioxidants such as vitamin E and alpha-lipoic acid are reported to be effective in protecting animals from oxidative damage induced by AFB_1_, so SSGL may have similar abilities. Indeed, lower oxidative status and higher antioxidant capacity were observed in broilers fed on a diet supplemented with SSGL in the present study, indicating that dietary SSGL could protect broilers from oxidative damage induced by AFB_1_.

In the present study, the results showed that dietary SSGL significantly increased the contents of serum TP, IgA and IgG, and decreased the AFB_1_-induced toxins to serum immunity. Mohan et al. [54] observed that dietary polysaccharides of *G. lucidum* significantly increased the muscle TP of *Macrobrachium rosenbergii*. Finisher pigs fed diets that received polysaccharides of *G. lucidum* had higher contents of serum TP, globulin and IgG [55]. These results suggested that *G. lucidum* may have the ability to increase the protein synthesis of animals. Di et al. [56] found that the high-performance thin-layer chromatography fingerprint profiles of carbohydrate and acid hydrolyzates of polysaccharides from *G. lucidum* and the spores were quite similar, indicating that they may have similar bioactivities. The (1 → 3)-α-d-glucan of SGL enhanced the T and B lymphocyte proliferation and antibody production of rats [17]. Thus, the mechanism of SSGL improving serum IgA and IgG in this study could be partially due to the decrease in the inhibition of protein synthesis induced by AFB_1_.

## 4. Conclusions

The results from this study demonstrated that diets contaminated with AFB_1_ (22.5–25.0 μg/kg) suppressed the growth performance, antioxidant capacity and immune action of broilers. However, the addition of SSGL significantly counteracted the adverse effects of AFB_1_, effectively improving growth performance and reducing the oxidative stress and immunosuppression of broilers; so SSGL, as a feed additive for inhibiting aflatoxicosis, may have promising potential in feed industrial applications.

## 5. Materials and Method

### 5.1. Sporoderm-Broken Spores of Ganoderma lucidum

SGL in present study was supplied by Riverside Ganoderma Lucidum Planting Co. Ltd. (Xiuyan Manchu Autonomous County, Anshan, Liaoning Province, China). The spores was dried at 55 °C for 24 h, and then broken by supercritical fluid extraction device (Nantong Hua′an Co. Ltd., Nantong, China). Briefly, approximately 150 g of spores was loaded into a steel cylinder equipped with mesh filters (6.5 μm) on both ends. Liquefied CO_2_ was pumped into the vessel and the pressure was raised to 35 MPa consequently. The process was last for 4 h and the temperature was set at 25 °C by the temperature controller. At the end of process, the pressure was quickly released within 1 min, and the spores was broken due to rapid depressurization. The SSGL were removed and stored at −20 °C before adding into the diets [12,57].

### 5.2. Birds, Diets and Management

This study was approved by the Animal Care and Use Committee of the China Agricultural University (ethical approval code: CAU20151028-2; Date: 28 October 2015). A total of 300 one-day-old male Arbor Acre broiler chickens were obtained from a commercial company (Beijing Huadu Yukou Poultry Co., Ltd., Beijing, China). Birds with similar body weights (40.0 ± 1.0 g) were randomly assigned to four treatments with five replicate pens of 15 birds per pen. The diets for treatments were: Control (the basal diets containing 20% normal peanut meal in the starter and 18% in the finisher period, without any mycotoxins); AFB_1_ (moldy peanut meal naturally contaminated with 121.7 μg/kg AFB_1_ substituting for all the normal peanut meal in the basal diet); SSGL (basal diet received 200 mg/kg of SSGL); AFB_1_+SSGL (AFB_1_ diet supplemented with 200 mg/kg of SSGL). The contents of mycotoxins (AFB_1_, AFB_2_, AFG_1_, AFG_2_, deoxynivalenol (DON), zearalenone (ZEA) and ochratoxin (OTA)) in basal diet and AFB_1_-contaiminated diets were tested with HPLC (Shimadzu LC-10 AT, Shimadzu, Tokyo, Japan) method [25,58]. Briefly, 25 g of the milled samples were mixed with 80 mL of methanol-water (80:20 *v*/*v*) for aflatoxins; water for DON; acetonitrile-water (70:30 *v*/*v*) for ZEA; methanol-water (60:40 *v*/*v*) for OTA, the mixtures were shaken for 2 h. The extracts were filtered, and the filtrate was cleaned up through an immunoaffinity column (Vicam, Milford, MA, USA) before HPLC determination. The limits of detection (LOD) and quantification (LOQ) were: 0.10 and 0.50 μg/kg for AFB_1_; 0.08 and 0.25 μg/kg for AFB_2_; 0.15 and 0.50 μg/kg for AFG_1_; 0.10 and 0.50 μg/kg for AFG_2_; 1.00 and 5.00 μg/kg for ZEA; 10.00 and 50.00 μg/kg for DON; 0.21 and 0.60 μg/kg for OTA, respectively. The final contents of AFB_1_ in diets received moldy peanut meal (AFB_1_ and AFB_1_+SSGL groups) were 25.0 ± 1.2 μg/kg in starter period and 22.5 ± 1.1 μg/kg in finisher period; the other mycotoxins were determined to be at concentrations below detection limits. Hardly any aflatoxins or other mycotoxins were found (below detection limits) in basal diets.

The formulation of the basal diets are presented in Table 5, all essential nutrients in the basal diet met NRC (1994) [59]. The feeding trial period lasted for 44 days.

The trial was conducted in two periods consisting of a starter period from day 1 to 21 and a finisher period from day 22 to 44. The rearing system was designed as flooring rearing. All birds were raised in weird-floored pens (100 × 100 × 60 cm) in an environmentally controlled room with continuous light. The temperature was maintained at 35–37 °C (65% relative humidity) for the first two days, and 33–35 °C for day 3–7. The room temperature was gradually decreased by 2 °C per week until to 24 °C, and then maintained unchanged. Water and diets were provided ad libitum. All birds were inoculated with Newcastle disease vaccine on day 7 and day 21 and inoculated with infectious bursa disease vaccine on day 14 and day 21.

### 5.3. Sample Collection

At 44 days of age, one bird close to the average weight was selected from each pen. After the birds were fasted for 12 h, blood sample was collected in tubes (without anticoagulant) from a wing vein with a 5 mL syringe. The samples were centrifuged at 1000× *g* for 10 min, and the serum was separated and then stored at −20 °C until analysis. The chickens were then sacrificed; the liver and spleen were removed immediately. A portion of the liver and the whole spleen were snap frozen in liquid nitrogen and stored at −70 °C.

### 5.4. Oxidative Stress Indices in Liver and Spleen

Liver and spleen tissues (about 1 g) were cut into small pieces and homogenized in ice-cold saline buffer (1:9, *wt*/*v*) using an Ultra-turrax (T8, IKA-labortechnik, Staufen, Germany) to form homogenate at a concentration of 0.1 g/mL for further analysis. The homogenate was centrifuged at 1000× *g* for 10 min at 4 °C, and the supernatant was collected and stored in freezer for assays of T-AOC, CAT, GR, GSH-PX, GSH, MDA, LPO, H_2_O_2_, hydroxyl radical scavenger activity (HRSA) and TP. All the assays were measured with commercial assay kits (Nanjing Jiancheng Bioengineering Institute, Nanjing, China) and the procedures accordingly.

### 5.5. Serum Concentrations of Total Protein and Immunoglobulins

The contents of serum TP, IgA, IgM and IgG were measured using commercial kits from Nanjing Jiancheng Bioengineering Institute. The measurements were performed according to the detection kit instructions.

### 5.6. Statistical Analyses

All data were subjected to analysis by one-way ANOVA using SAS (Version 8e, SAS Institute, Cary, NC, USA). Duncan’s multiple range test was used for multiple comparisons when a significant difference was detected. Means were considered significantly different at *p* < 0.05.

## Figures and Tables

**Table 1:** Effects of SSGL on growth performance of male Arbor Acre broiler chickens exposed to a low level of AFB_1_.

Index ^1^	Control	AFB_1_	SSGL	AFB_1_+SSGL	SEM	*p*-Value
0–21 d						
ADFI (g/d)	53.0 ^a^	51.0 ^b^	52.2 ^ab^	53.0 ^a^	0.296	0.039
ADG (g/d)	33.5	32.9	33.3	33.1	0.141	0.441
F:G	1.58	1.55	1.57	1.60	0.011	0.425
22–44 d						
ADFI (g/d)	135.7 ^a^	132.0 ^b^	138.0 ^a^	137.9 ^a^	0.776	0.008
ADG (g/d)	70.4 ^b^	67.8 ^c^	74.0 ^a^	73.8 ^a^	0.632	<0.001
F:G	1.93 ^ab^	1.95 ^a^	1.87 ^b^	1.87 ^b^	0.012	0.011
0–44 d						
ADFI (g/d)	98.1 ^a^	95.2 ^b^	99.0 ^a^	99.4 ^a^	0.515	0.006
ADG (g/d)	53.6 ^b^	51.9 ^c^	55.5 ^a^	55.3 ^a^	0.360	<0.001
F:G	1.83 ^a^	1.84 ^a^	1.78 ^b^	1.80 ^ab^	0.008	0.069

^1^ Data are expressed as group mean values; ADFI, average daily feed intake; ADG, average daily gain; F:G, feed:gain ratio, equal to ADFI/ ADG; SEM, standard error of the mean; ^a–c^ Means within the same row with different superscripts are significantly different (*p* < 0.05).

**Table 2:** Effects of SSGL on oxidative status in liver and spleen of male Arbor Acre broiler chickens exposed to a low level of AFB_1_.

Index ^1^	Control	AFB_1_	SSGL	AFB_1_+SSGL	SEM	*p*-Value
Liver						
H_2_O_2_ (mmol/g prot)	10.74 ^c^	15.06 ^a^	9.60 ^d^	12.38 ^b^	0.540	<0.001
MDA (nmol/g prot)	2.57 ^b^	3.10 ^a^	2.07 ^d^	2.26 ^c^	0.102	<0.001
LPO (μmol/g prot)	0.82 ^b^	1.02 ^a^	0.82 ^b^	0.85 ^b^	0.024	<0.001
Spleen						
H_2_O_2_ (mmol/g prot)	14.38 ^b^	16.19 ^a^	14.18 ^b^	14.23 ^b^	0.252	0.001
MDA (nmol/g prot)	1.87 ^ab^	1.95 ^a^	1.80 ^b^	1.84 ^ab^	0.019	0.046
LPO (μmol/g prot)	4.20 ^b^	5.08 ^a^	3.34 ^c^	3.04 ^d^	0.207	<0.001

^1^ Data are expressed as group mean values; H_2_O_2_, hydrogen peroxide; MDA, malondiadehyde; LPO, lipid peroxidation. SEM, standard error of the mean; ^a–d^ Means within the same row with different superscripts are significantly different (*p* < 0.05).

**Table 3:** Effects of SSGL on antioxidant defense system in liver and spleen of male Arbor Acre broiler chickens exposed to a low level of AFB_1_.

Index ^1^	Control	AFB_1_	SSGL	AFB_1_+SSGL	SEM	*p*-Value
Liver						
T-AOC (U/mgprot)	2.19 ^b^	2.04 ^c^	2.32 ^a^	2.28 ^ab^	0.032	<0.001
CAT U/mgprot)	8.13 ^b^	7.35 ^c^	9.43 ^a^	8.05 ^b^	0.205	<0.001
T-SOD (U/mgprot)	16.95	16.78	16.12	16.58	0.227	0.647
GR (U/gprot)	4.47 ^b^	3.74 ^c^	6.74 ^a^	4.84 ^b^	0.297	<0.001
GSH-Px (U)	43.25 ^a^	37.50 ^b^	43.49 ^a^	36.63 ^b^	0.853	<0.001
GSH (mg/gprot)	2.03 ^b^	2.07 ^b^	2.43 ^a^	2.44 ^a^	0.058	0.001
HRSA (U/g prot)	2.42 ^a^	1.98 ^b^	2.58 ^a^	2.45 ^a^	0.062	<0.001
Spleen						
T-AOC (U/mgprot)	2.46 ^b^	1.78 ^c^	3.28 ^a^	2.52 ^b^	0.139	<0.001
CAT (U/mgprot)	5.35 ^a^	4.33 ^b^	5.42 ^a^	4.48 ^b^	0.131	<0.001
T-SOD (U/mgprot)	22.22 ^a^	19.27 ^b^	22.11 ^a^	21.64 ^a^	0.363	0.001
GR (U/gprot)	6.34 ^c^	6.02 ^c^	9.25 ^a^	7.52 ^b^	0.346	<0.001
GSH-Px (U)	47.73 ^a^	43.39 ^b^	47.59 ^a^	44.94 ^b^	0.542	<0.001
GSH (mg/gprot)	3.92 ^b^	3.64 ^c^	4.32 ^a^	4.36 ^a^	0.079	<0.001
HRSA (U/g prot)	1.87 ^c^	1.61 ^d^	2.48 ^a^	2.06 ^b^	0.084	<0.001

^1^ Data are expressed as group mean values; T-AOC, total antioxidant capability; CAT, catalase; GR, glutathione reductase; GSH-Px, glutathione peroxidase; GSH, reduced glutathione; HRSA, Hydroxyl radical scavenger activity; SEM, standard error of the mean. ^a–d^ Means within the same row with different superscripts are significantly different (*p* < 0.05).

**Table 4:** Effects of SSGL serum immunoglobulins of male Arbor Acre broiler chickens exposed to a low level of AFB_1_.

Index ^1^	Control	AFB_1_	SSGL	AFB_1_+SSGL	SEM	*p*-Value
TP (g/L)	24.82 ^a^	22.73 ^b^	25.94 ^a^	25.07 ^a^	0.374	0.007
IgA (g/L)	0.291 ^a^	0.284 ^b^	0.298 ^a^	0.295 ^a^	0.002	0.003
IgG (g/L)	0.266 ^a^	0.232 ^b^	0.273 ^a^	0.257 ^a^	0.005	<0.001
IgM (g/L)	0.224	0.225	0.229	0.236	0.002	0.288

^1^ Data are expressed as group mean values; TP, total protein; SEM, standard error of the mean; ^a,b^ Means within the same row with different superscripts are significantly different (*p* < 0.05).

**Table 5:** Ingredients and nutrient composition of the basal diet.

Ingredient (%)	Starter Period (0–21 d)	Finisher Period (22–44 d)
Corn	57.95	60.79
Soybean meal	14.20	13.30
Peanut meal	20.00	18.00
Limestone	1.30	1.00
Dicalcium phosphate	1.80	1.50
Salt	0.30	0.30
Soybean oil	3.00	4.00
Lysine [98.5%]	0.46	0.34
DL-Methionine	0.37	0.20
Threonine	0.19	0.14
Choline chloride	0.10	0.10
Vitamin premix ^1^	0.03	0.03
Mineral premix ^2^	0.30	0.30
Total	100.000	100.00
Nutrition componen		
CP	21.48	20.03
ME(Mcal/kg)	2.99	3.09
Ca	1.01	0.82
Total phosphorus	0.67	0.61
Lys	1.15	1.01
Met	0.63	0.45

^1^ Provided per kilogram of diet: vitamin A, 15,000 IU; cholecalciferol, 3000 IU; vitamin E, 20 IU; vitamin K 3, 2.18 mg; thiamine, 2.15 mg; riboflavin, 8.00 mg; pyridoxine, 4.40 mg; vitamin B 12, 0.02 mg; Calcium pantothenate, 25.60 mg; nicotinic acid, 65.80 mg; folic acid, 0.96 mg; biotin, 0.20 mg. ^2^ Provided per kilogram of diet: Fe, 100.0 mg; Cu, 8.0 mg; Zn, 78.0 mg; Mn, 105.0 mg; I, 0.5 mg; Se, 0.3 mg.
